# Prevalence and factors associated with potential substance use disorders among police officers in urban Tanzania: a cross-sectional study

**DOI:** 10.1186/s12888-023-04663-6

**Published:** 2023-03-16

**Authors:** Harrieth P. Ndumwa, Belinda J. Njiro, Joel M. Francis, Thomas Kawala, Charles J. Msenga, Ezekiel Matola, Juhudi Mhonda, Hillary Corbin, Omary Ubuguyu, Samuel Likindikoki

**Affiliations:** 1 School of Medicine, Muhimbili University of Health and Allied Sciences, Dar Es Salaam, Tanzania; 2 Department of Family Medicine and Primary Care, School of Medicine, University of the Witwatersrand, Johannesburg, South Africa; 3 Department of Epidemiology and Biostatistics, School of Public Health and Social Sciences, Muhimbili University of Health and Allied Sciences, Dar Es Salaam, Tanzania; 4 Medical Service Unit, Tanzania Police Force, Dar Es Salaam, Tanzania; 5 Directorate of Curative Services, Ministry of Health, Dodoma, Tanzania

**Keywords:** Substance use disorder, Alcohol, Tobacco, Police officers, Tanzania

## Abstract

**Background:**

Substance Use Disorders (SUDs) among Police Officers has been a concern to many professionals in the field of health, research and criminal justice since their work is subjected to higher levels of stress and hence more likely to use alcohol or tobacco as a coping mechanism. However, little is known about SUDs among Police Officers in Tanzania. Therefore, we assessed the prevalence and factors associated with SUDs among Police Officers in urban Tanzania.

**Materials and methods:**

A cross-sectional study was conducted between April and October 2019 among Police Officers in Dar es Salaam. Multistage cluster sampling technique was used to recruit study participants. The WHO-Alcohol, Smoking and Substance Involvement Screening Test (ASSIST) version 3.0 was used to measure potential SUDs. Bivariate and multivariate analyses were performed to establish associations between potential SUDs and predictors of interest, and an alpha of 5% was used in sample size calculation.

**Results:**

A total of 497 participants were enrolled, of these, 76.6% (376/491) were males, the median age (years) and IQR was 37.0 (30.0, 47.0). The prevalence of past three months use of alcohol and tobacco were 31.3% and 6.3%, respectively. About 13.3% (62/468) and 6.2% (29/468) of Police Officers met criteria for potential Alcohol Use Disorder (AUD) and potential Tobacco Use Disorder (TUD) respectively. In adjusted analysis, participants with depression had about two times increased odds for potential AUD (aOR: 2.27, 95% CI; 1.12 – 4.58, *p* = 0.023) than those with no depression. Potential AUD and depression were associated with about eight times (aOR: 8.03, 95% CI; 3.52 – 18.28, *p* < 0.01) and more than twice (aOR: 2.63, 95% CI; 1.12 – 6.15, *p *= 0.026) higher odds for potential TUD respectively.

**Conclusion:**

Substance use and potential substance use disorders particularly AUD and TUD are common among Police Officers in urban Tanzania. Depression was found to be an important factor for potential AUD and TUD among Police Officers and, a significant co-occurrence of potential AUD with potential TUD was observed. Findings from this study call for interventions, for example, the need to routinize the brief motivational interview services for alcohol and tobacco use among Police Officers.

## Introduction

Police Officers are exposed to life-threatening situations, fatal accidentsand injuries resulting from violence and domestic disputes more frequently than other professions [1–3]. Police Officers' response to various events can lead to psychologically, cognitively and physically debilitating conditions that impair their ability to function and may lead to substances use for coping [4]. Substance Use Disorders among Police Officers is common for a range of reasons including coping with job and life stressors, mistreatment of pain, dealing with anxiety, depression, and post-traumatic stress disorder (PTSD) [1, 2].

Substance Use Disorders (SUDs) contribute significantly to the global burden of morbidity and mortality [5]. The United Nations Office on Drugs and Crime (UNODC) estimated that about 167,000 deaths which occurred in 2017 were related to SUDs and lead to about 21 million Disability Adjusted Life years (DALYs) [6]. The burden of SUDs extends beyond health to further impact social and economic status of the affected individuals and families [5]. The World Drug Report 2020 highlights important socioeconomic characteristics associated with SUDs including poverty, violence, low social support, income inequality and crime [7]. Besides being a leading cause of lung cancer [8], tobacco smoking resulted in the loss of over 2.1 million life years (2.9%) [9]. Further, alcohol use is an underlying cause of more than 30 conditions including infectious diseases, cardiovascular diseases and neuropsychiatric diseases [10].

The prevalence of SUDs varies significantly around the world with the highest prevalence (16%) found in Eastern and Central Europe [11]. Globally, 31 million persons have SUDs with 11 millions of these people injecting drugs [12]. The South African Stress and Health (SASH) study on patterns of substance use shows the cumulative prevalence of substance use in the general population to be 38% for alcohol use, 30% for tobacco smoking, 8.4% for cannabis use and 2.0% for other drugs [13]. South-East Asia and Western Pacific are found to have a prevalence of 10% and 13% respectively for Alcohol Use Disorder (AUD) [11]. In the United States, the prevalence of AUD was found to be 10% with higher rates of alcohol consumption found among males (34.6%) as compared to females (15.6%) [11]. In Tanzania, males in the general population have a prevalence of around 11–28% for AUD [14, 15]. An overall prevalence of AUD in the general population of Tanzania is 6.8% [15] whereas the prevalence of tobacco use is 8.7% of [16].

A study done in the U.S by Miller et al. found that, about one in every six Police Officers required substance use intervention and one in every twenty had an untreated AUD [1]. Moreover, 7.8% of Police Officers in the U.S met criteria for lifetime alcohol abuse or dependence with 18.1% of males and 15.9% of female Police Officers having experienced adverse consequences from alcohol use [17]. In urban Tanzania, the prevalence of alcohol use among Police Officers was found to be 14.8% with males having a higher proportion 38.8% as compared to females 12.8% [18].

Previous studies have reported higher rates of tobacco use and TUD among police officers compared to other professionals [19, 20]. Madu et al. found a prevalence of smoking among police officials in South Africa to be 37.4% [20]. In Poland, police officers were found to have about three times increased odds for TUD as compared to other civil workers [21]. Moreover, tobacco use among Police Officers in Uganda was found to be about five times the general public (25.5% vs 5.3%) [22].

Police Officers are reported to have an increased vulnerability for violence, depressive symptoms, suicidality, Post Traumatic Stress Disorders and HIV risky behaviors [23–25]. A high vulnerability of SUD among police officers has been less studied in low resource settings such as Tanzania. It is therefore imperative to investigate SUDs among Police Officers which will help to see the need for integrating SUDs in the pre-existing interventions for risky behaviors in this sub-population. Considering Police Officers’ roles in maintaining public peace, law and order in society as well as to prevent, detect and combat crime [26] it is paramount to protect their physical and mental well-being. This study aimed at assessing the prevalence and factors associated with SUDs particularly AUD and TUD among Police Officers in urban Tanzania. Findings of this study will help in strengthening the existing system to address substance use and integrate with existing interventions for violence, suicide and HIV risky behaviors so as to improve the overall physical and mental well-being of Police Officers in the country.

## Materials and methods

### Study design, setting and duration

This was a cross-sectional survey conducted among Police Officers in Dar es Salaam, Tanzania. This study was conducted using quantitative data collection method from April to October 2019. Dar es Salaam is largest city in the country with the biggest port along the Indian ocean. The city is the business capital, estimated to have a population of 6.4 Million and an annual growth rate of 5.6 percent [27].

### Study population, inclusion and exclusion criteria

**Fig. 1:**
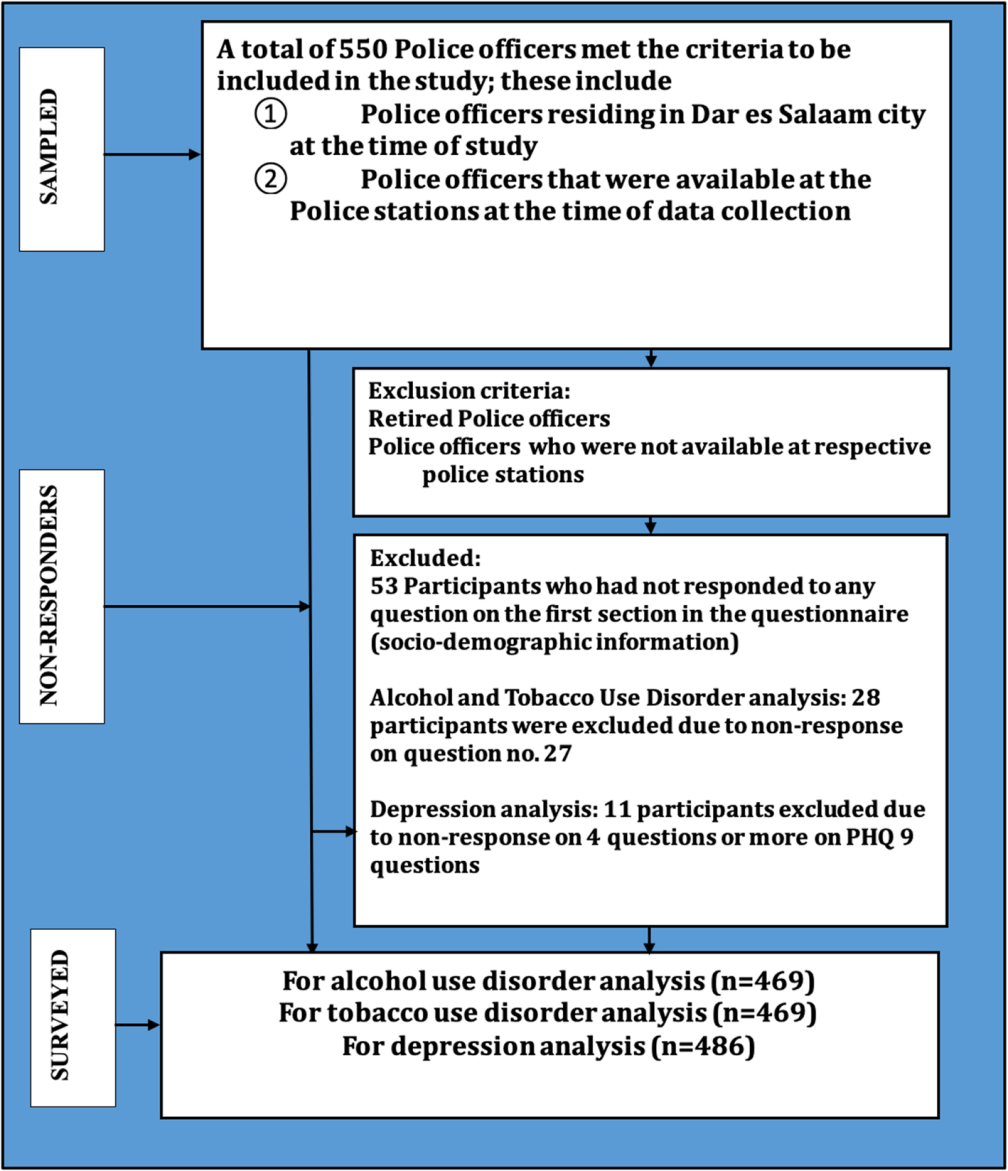
Participants’ recruitment flow chart: PHQ-9, Patient Health Questionnaire-9

The police force in Tanzania is a national body within the Ministry of Home Affairs and is lead by the Inspector General of Police. The Tanzania Police Force is divided into five departments, which fall under the control of individual Commissioners. Police Officers are recruited through an interview process after making applications and fulfilling the required criteria. After recruitment, they undergo a mandatory police training in specialized police colleges located in different regions of the country [28]. The study population was composed of Police Officers who were residing in Dar es Salaam Police Zone and were available in their respective Police stations at the time of study. Retired police officers and those who were absent at the respective police stations during the study period were excluded. No age restriction was considered during the enrolment **(**Fig. 1**).**

### Sample size determination and technique

A minimum sample size of 497 Police Officers was calculated using the proportional formula for calculating sample size (Kish and Leslie formula) [29]. The expected proportion for substance use of 19.3%, obtained from a study done among Police Officers in Kampala, Uganda by Ovuga et al. [18] and an alpha of 5% were used for calculation. Multistage cluster sampling involving three stages was used to obtain the sample. Multistage cluster sampling is an approach best used to obtain a sample in a complex population [30]. The first stage involved selection of all three Police Regions in Dar es Salaam Police Zone. The second stage involved random selection of two Police Districts / Units from each of the three Police Regions. No sampling was done at the selected unit level, all available officers were invited to participate in the study. Prior communication through phone calls was made to the heads of Police Officers where data was to be collected so as to remind Police Officers to be available at their work areas at the time of data collection. We conducted data collection during the morning hours where most police officers report at their respective stations before departing to their allocated work sites. All Police Officers meeting our inclusion criteria, available at their areas of work during the data collection period and consented to participate in the study were included.

### Data collection process and instruments

Data collection was conducted by HPN, BJN, TK, EM, JM, HC and two trained research assistants. Two District Police stations in each of the 3 Police Regions and 3 special Police Units (Field force unit-FFU, Police Band and Information and communication technology-ICT) making a total of 9 sites took part in the data collection process. Data collection involved all consenting Police Officers available at the time of recruitment. In all visited sites, participants were gathered in a room where privacy was assured, explained briefly on the purpose of the study and after consenting to participate, they were given questionnaires and oriented on how to respond to the set of questions in the tool.

A structured self-administered questionnaire that included socio-demographics (age, sex, marital status, education, rank, years served in police force, promotion status, police section, operational status, years served in police section, police region), social support, depression and substance use questions was used. The questionnaire had four main sections; socio-demographic information with thirteen items, social support with twelve items, depression with nine items and substance use with nine items. The social support questionnaire was only available in English version, it was therefore translated into Swahili language and incorporated into other sections of the questionnaire. The administered questionnaire was in Swahili, a national language spoken by many in the country.

### Study variables and their measures

Screening for substance use and SUDs was done using the Alcohol, Smoking and Substance Involvement Screening Test developed by the WHO (WHO-ASSIST) version 3.0. This tool was developed by an international group of substance abuse researchers to detect and manage substance use and related problems in primary care settings and in other target groups considered to be at high risk of substance related problems [31]. The ASSIST tool is found to accurately identify tobacco, alcohol, and cannabis use disorders at a sensitivity of 95%-100% and specificity of 79%-93%, in this study the participants classified as medium and high risk were compared with those classified as low risk [32]. Comparable sensitivity and specificity is also noted for diverse subgroups of patients in terms of gender, age, race/ethnicity, marital status, educational levels, and post traumatic stress disorder status [33]. The tool has a total of 9 questions in which the first 7 questions had 10 responses from i-j corresponding to tobacco products, alcoholic beverages, cannabis, stimulants, inhalants, sedatives, hallucinogens opioids and other substances respectively. The 8^th^ and 9^th^ questions were based on patterns of injecting drugs. The score for each substance in the WHO-ASSIST ranges from 0, which is the minimum score to 44 which is the maximum score. For all substances except alcohol, a score ranging from 0–3 indicates a low risk level, a score ranging from 4–26 indicates a moderate risk level and a score from 27 + indicates a high-risk level of substance use. For alcohol use, a score of 0 – 10 indicates a low risk level, 11 – 26 indicates a moderate risk level and, 27 + indicates a high-risk level. Due to a small sample size, high and moderate risk for substance use was combined to represent a potential substance use disorder (AUD and TUD), moreover, both high and moderate risk of substance use requires substantial follow up and management [31, 32]. Additionally, we used the substance use question in the tool, “In the past three months, how often have you used the substances you mentioned” to establish the prevalence of substance use in this study.

Perceived social support was measured by the short version of the Interpersonal Support Evaluation List consisting of 12 items (ISEL-12). ISEL-12 has subscales designed to measure three dimensions of perceived social support namely appraisal, belonging and tangible supports. Each of these dimensions was measured by a 4-point Likert scale ranging from 1 = definitely false to 4 = definitely true, 6 questions were reverse scored. The tool has been previously validated in English and Hispanic communities with good internal consistency (overall Cronbach’s alpha above 0.70) however, the scores were inadequate for the appraisal (α = 0.65), belonging (α = 0.62), and tangible (α = 0.57) subscales; test–retest reliability was 0.88–0.90 [34, 35]. In this study, the internal consistency (Cronbach’s alpha) for the ISEL-12 tool was 0.69 with the internal reliability scores of 0.52 for the appraisal support subscale, 0.37 for the belonging support subscale and 0.25 for the tangible support subscale. Scores of 12–30, 31–41, and 42–48 were defined as low, fair and high social support respectively [35]. Perceived social support was however excluded in the logistics regression analysis due to low internal reliability of the subscales.

Depression was assessed using a validated Patient Health Questionnaire-9 (PHQ-9) consisting of 9 items. The reliability of PHQ-9 has been documented in Tanzanian setting with a Cronbach’s alpha, α = 0.83 [36]. In this study, the internal consistency for the PHQ-9 tool was 0.84. Participants were required to choose if over the last 2 weeks, they have experienced the listed symptoms. The responses were either 0 = not at all, 1 = for several days, 2 = more than half the days, or 3 = nearly every day; depression was defined by a score of > 9, with a previously reported sensitivity and specificity of 78% and 87% respectively [36].

### Data management and analysis

All data entry, cleaning and subsequent analysis were completed using SPSS for Windows Version 20. Descriptive statistics for demographic characteristics and prevalence of potential SUDs were summarized using frequencies, percentages, median and the corresponding interquartile range (IQR). Bivariate and Multivariate logistic regression analyses were computed and the odds ratios with the corresponding 95% Confidence Interval (CI) were used to determine associations between socio-demographic factors, perceived social support, depression, potential AUD and potential TUD. Multivariate logistic regression analysis models included all factors with a *p* value of < 0.20 in bivariate analysis. *A p-value* of < 0.05 was considered to be statistically significant.

## Results

### Demographic characteristics of study participants

A total of 550 participants were enrolled in the study and only 497 completed the survey, the response rate was 90.4%. Fifty-three participants had incomplete information in questionnaires and were not included in the final analyses. Majority of the participants were males (76.6%, 376/491). About 4 out of 10 (38.1%, 151/397) participants aged 41 years or older, the median age (interquartile range) was 37.0 [16] years. About three-quarters (76.3%, 371/473) of participants were married and about a half (47.0%, 231/491) reported having attained secondary level of education. Just above half 52.6% (249/473) of the participants reported to have worked in the Police force for 5–14 years. An overall majority 83.4% (412/494) of the participants were serving as the Rank-and-File Police Officers. Just above a half (54.8%, 268/489) of participants reported to have been working in the specialized duties and about three quarters (74.3%, 355/489) reported to have engaged in different operational activities (Table 1)**.**

**Table 1:** Socio-demographic characteristics of Police Officers in Urban (Dar Es Salaam), Tanzania 2019

Characteristic	*N*	%
**Sex (** ***n*** ** = 491)**		
Male	376	76.6
Female	115	23.4
**Age groups (** ***n*** ** = 397)**		
21–30	105	26.4
31–40	141	35.5
41 and above	151	38.1
**Marital status (** ***n*** ** = 473)**		
Single	92	19.4
Married	371	76.3
Separated and widowed	20	4.3
**Education (** ***n*** ** = 491)**		
Primary	53	10.8
Secondary	231	47.0
Higher education	207	42.2
**Years served in Police Force (** ***n*** ** = 473)**		
5–14	249	52.6
15 and above	224	47.4
**Last year of promotion (** ***n*** ** = 479)**		
< 5 years	344	71.8
5–10 years	97	20.3
> 5 years	38	7.9
**Rank at work (** ***n*** ** = 494)**		
Gazette Officer ^a^	22	4.5
Inspector ^b^	60	12.1
Rank and file ^c^	412	83.4
**Police Sections (** ***n*** ** = 489)**		
General duty	130	26.6
Specialized sections ^d^	359	73.4
**Years served in Police Section (** ***n*** ** = 474)**		
One year and below	45	9.5
More than a year	114	24.0
More than five years	315	66.5
**Operational activities (** ***n*** ** = 478)**		
Yes	355	74.3
No	123	25.7
**Perceived Social Support (** ***n*** ** = 426)**		
High	163	38.3
Fair	215	50.4
Low	48	11.3
**Police Regions (** ***n*** ** = 497)**		
Ilala	126	25.4
Kinondoni	127	25.6
Temeke	86	17.3
FFU	76	15.3
Police Band	44	8.9
Main Store	38	7.6

^*a*^*Gazette Officers (Inspector General of Police-IGP, Commissioner of Police-CP, Deputy commissioner of Police-DCP, Senior Assistant Commissioner of Police-SACP, Assistant Commissioner of Police-ACP, Senior Superintendent of Police-SSP)*

^*b*^*Inspectors (Superintendent of Police-SP, Assistant Superintendent of Police-ASP).*

^*c*^*Regimental Sergeant Majors/Sergeant majors (RSM/SM), Station Sergeant (SSG), Sergeant (SGT), Corporal Police (CPL) and Police Constable (PC).*

^*d*^*Field Force Unit (FFU), Information and Communication Technology (ICT), Medical Unit, Criminal Investigation Department (CID), Main Police Depot, Traffic Department, Police Band, Police Colleges, Dogs and horse section, Mechanical department, Anti-drugs Unit, Construction and building department*

### Prevalence of substance use and potential substance use disorders among police officers

About 4 out of 10 (40.2%, 200/497) and about a third (31.4%, 156/497) of participants reported to have ever and recently used one or more substances, respectively. The most common recently used substance was alcohol (31.3%, 147/469) followed by tobacco (6.2%, 29/469). Only 10 out of 469 participants reported recent use of one or more of the following substances; cannabis, cocaine, amphetamine, inhalants, sedatives, hallucinogens and opioids.

About 1 in 10 (13.2%, 62/468) participants were found to have potential AUD (combined high and moderate risk; 10.3% had moderate risk and 3.0% had high risk) whereas 6.2% (29/469) reported recent use of tobacco had potential TUD (combined high and moderate risk; 5.1 had moderate risk and 1.1 had high risk). The prevalence of potential sedatives use disorder and cannabis use disorder were found to be 1.5% (7/465) and 1.1% (5/467) respectively (Table 2).

**Table 2:** Prevalence of potential substance use disorders among Police Officers in urban (Dar Es Salaam), Tanzania 2019

Characteristic	*N*	%
**Tobacco (** ***n*** ** = 468)**		
Low	439	93.8
High	29	6.2
**Alcohol (** ***n*** ** = 468)**		
Low	406	86.8
High	62	13.3
**Cannabis (** ***n*** ** = 467)**		
Low	462	98.9
High	5	1.1
**Cocaine (** ***n*** ** = 466)**		
Low	465	99.8
High	1	0.2
**Amphetamine (** ***n*** ** = 467)**		
Low	463	99.1
High	4	0.9
**Inhalants (** ***n*** ** = 467)**		
Low	467	100.0
**Sedatives (** ***n*** ** = 465)**		
Low	458	98.5
High	7	1.5
**Hallucinogens (** ***n*** ** = 467)**		
Low	466	99.8
High	1	0.2
**Opioids (** ***n*** ** = 468)**		
Low	466	99.6
High	2	0.4

*High and moderate risk for substance use is combined representing substance use disorders*

### Factors associated with potential alcohol use disorder among police officers

Bivariate analysis indicated that sex, age groups and depression were statistically significant factors for potential AUD among participants. However, in multivariate logistics regression model, depression was found to be significantly associated with AUD among participants. Police Officers with depression had two times increased odds for potential AUD (aOR: 2.27, 95% CI; 1.12 – 4.58, *p* = 0.023) compared to those without depression (Table 3)**.**

**Table 3:** Bivariate and Multivariate Analysis of factors associated with Potential Alcohol Use Disorder among Police Officers in urban (Dar Es Salaam), Tanzania 2019

Characteristic/variables	Alcohol (n, %)	cOR (95% CI)	*p-value*	aOR (95% CI)	*p-value*
**Sex (** ***n*** ** = 464)**					
Male	54 (15.2)	2.27 (1.04 – 4.92)	**0.039**	1	
Female	8 (7.3)	1		0.42 (0.17 – 1.01)	0.052
**Age groups (** ***n*** ** = 375)**					
21–30	7 (7.1)	1		1	
31–40	21 (15.6)	2.40 (0.98 – 5.88)	0.057	2.21 (0.81 – 6.07)	0.123
41 and above	25 (17.6)	2.78 (1.51 − 6.71)	**0.023**	2.23 (0.60 – 8.29)	0.232
**Marital status (** ***n*** ** = 446)**					
Single	8 (9.5)	1		1	
Married	49 (14.3)	1.59 (0.72 – 3.50)	0.250	1.10 (0.39 – 3.10)	0.854
Others	5 (25.0)	3.17 (0.91 − 11.02)	0.070	2.42 (0.47 – 12.46)	0.292
**Education (** ***n*** ** = 464)**					
Primary education	8 (16.3)	1			
Secondary education	25 (11.5)	0.66 (0.28 – 1.58)	0.353	-	-
Higher education	29 (14.7)	0.86 (0.38 – 2.08)	0.779	-	-
**Years at work (** ***n*** ** = 450)**					
5–14	27 (11.4)	1		1	
15 and above	35 (16.4)	1.53 (0.89 − 2.63)	0.123	1.32 (0.51 – 3.40)	-
**Operational activities (** ***n*** ** = 453)**					
Yes	49 (14.6)	1		-	
No	12 (10.3)	0.67 (0.34 − 1.31)	0.240	-	-
**Police section (** ***n*** ** = 462)**					
General duty	15 (12.1)	1		-	
Specialized sections	47 (13.9)	1.17 (0.63 − 2.19)	0.614	-	-
**Depression (** ***n*** ** = 496)**					
No	96 (19.8)	1		1	
Yes	390 (80.2)	1.88 (1.02 – 3.43)	**0.042**	2.27 (1.12 – 4.58)	0.023*

The variables “Police rank” and “years last promoted” have been dropped from the table because of few numbers and therefore a risk of disclosure of study participants

^*^Factors that were statistically significant (*p* < 0.05)

*cOR* Crude Odds Ratio

*aOR* Adjusted Odds Ratio

### Factors associated with potential tobacco use disorder among male police officers

On bivariate and multivariate logistics regression analysis, depression and alcohol use disorder were found to be associated with potential TUD among male Police Officers. On multivariate logistics regression, male Police officers with AUD were found to have eight times increased odds of having potential TUD (aOR: 8.03, 95% CI; 3.52 – 18.28, *p* < 0.01) compared to those without AUD; those with depression had more than twice higher odds for potential TUD (aOR: 2.63, 95% CI; 1.12 – 6.15, *p* = 0.026) (Table 4).

**Table 4:** Bivariate and Multivariate Analysis of Factors associated with Tobacco Use Disorder among Male Police Officers in urban (Dar Es Salaam), Tanzania 2019

Characteristic/variables	Tobacco (n, %)	cOR (95% CI)	*p-value*	aOR (95% CI)	*p-value*
**Age group (** ***n*** ** = 375)**					
21–30	4 (4.1)	1		-	
31–40	11 (8.1)	2.09 (0.64 – 6.75)	0.221	-	-
41 and above	10 (7.0)	1.78 (0.54 – 5.85)	0.342	-	-
**Marital status (** ***n*** ** = 446)**					
Single	3 (3.6)	1		-	
Married	25 (7.3)	2.13 (0.63 – 7.23)	0.225	-	-
Others	1 (5.0)	1.42 (0.14 –14.4)	0.766	-	-
**Years at work (** ***n*** ** = 450)**					
5–14	14 (5.9)	1		-	
15 and above	15 (7.0)	1.21 (0.57 – 2.56)	0.625	-	-
**Operational activities (** ***n*** ** = 453)**					
Yes	22 (6.5)	1		-	
No	5 (4.3)	0.64 (0.24 – 1.72)	0.374	-	-
**Police section (** ***n*** ** = 462)**					
General duty	5 (4.0)	1		-	
Specialized sections	24 (7.1)	1.82 (0.68 – 4.88)	0.234	-	-
**Depression (** ***n*** ** = 486)**					
No	96 (19.8)	1		1	
Yes	390 (80.2)	3.12 (1.39 – 6.97)	**0.006**	2.63 (1.12 – 6.15)	0.026*
**Alcohol Use Disorder (** ***n*** ** = 468)**					
Yes	62 (13.3)	7.60 (3.46 – 16.71)			
No	406 (86.8)	1	** < 0.001**	8.03 (3.52 – 18.28)	< 0.001*

The variables “Police rank” and “years last promoted” have been dropped from the table because of few numbers and therefore a risk of disclosure of the study participants

^*^factors that were statistically significant (*p* < 0.05)

*cOR* Crude odds ratio

*aOR* Adjusted odds ratio

None of the females officers reported tobacco use

## Discussion

Our study found that substance use and substance use disorders particularly potential AUD and TUD are common among Police Officers in urban Tanzania. About 3 in every 10 Police Officers in urban Tanzania are using alcohol, a rate that is two times higher than that in the general population (17.2%) [16]. Most studies have linked higher rates of alcohol use among Police Officers with the stressful nature of their works and therefore use of alcohol and other substances as a coping mechanism [4]**.** On the other hand, 6.2% of Police Officers are found to use Tobacco, a rate that is relatively low as compared to 8.7% in the general population [16]. The prevalence of tobacco use in this study is also lower compared to a study done among Police Officers by Violanti et al. [37] and Copenhaver et al. [38]. The differences could be partly accounted for by the use of WHO-ASSIST tool in the current study as opposed to the Alcohol Use Disorders Identification Test (AUDIT) in the comparative studies.

In this study, about one out of ten participants (13.3%) met criteria for a potential Alcohol Use Disorder (AUD) which is almost two times that found in the general Tanzanian population (6.8%) [15] however lower compared to a study done among Police Officers in Uganda (19.2%) [39]. Male Police Officers were found to have a higher prevalence of potential AUD as compared to their female counterparts, consistent with studies done among Police Officers elsewhere [40–42]. The gender gap in the prevalence of potential AUD among Police Officers is lower compared to the general population [14, 15]. Furthermore, previous studies have reported that males who initiate binge alcohol drinking during adolescence have a higher likelihood of persistent binge drinking during adulthood as compared to females [43, 44] which could reflect a higher prevalence of potential AUD among policemen in this study. In this case, our findings suggest that policemen are assigned to more stressful jobs compared to police women [14, 45] and, the increased social vulnerability to alcohol consumption among males as compared to females which is also seen in the general population [46]. AUD in this study was found to be higher among older Police Officers, similar to Australian Police Officers [47]. These findings are possibly explained by increasing workload [42] and high risk of Post-Traumatic Stress Disorder (PTSD) as Police Officers are more likely to be exposed to more critical incidents with increasing age [38]. Higher prevalence of AUD is also observed in other studies [42, 48] and is likely to be caused by maladaptive coping mechanism to work challenges in the absence of social support [42]. Police Officers with depression were found to have significantly higher rates of AUD and TUD consistent with a study done by Fritz et al., which explains that SUD among Police Officers is a form of coping with depression and work stress [49].

The current study found 1 in every 10 Police Officers to have TUD. Less has been reported on TUD but rather tobacco use among Police Officers. A study done in Poland found that Police Officers have about three times increased odds for tobacco use as compared to other civil workers [21]. Brunault et al. found a significant association between tobacco use and PTSD among Police Officers [50]. Similar to alcohol use, tobacco use is also regarded as a method of coping with work stress [38] and debriefing following critical incidents among Police Officers [51]. Police Officers with AUD had significantly higher rates of TUD, this is consistent with findings from other studies and is explained by genetic response and neuroadaptation involved in regulating brain chemical systems such as tolerance and sensitization to both drugs [52, 53].

Generally, substance use and substance use disorder is found common among police officers as compared to the general population [18–20]. Alcohol use is particularly regarded as a part of police culture and norm as they are ostracized by the rest of the society members due to the nature of their work [20, 54]. Other studies have established lack of effective coping skills and recreational time influences use of substance among police officers [20]. Since occupational stress, exclusion from the rest of the society, post-traumatic stress disorder, depression, suicide and substance use appear to affect police officers [23–25], future studies should explore all these aspects using vigorous methodological approaches. Longitudinal study designs and mixed methods approaches will generate more information on substance use and establish a clear association between substance use, occupational, operational and organization factors. Through longitudinal studies, it will also be possible to establish causal relationship between SUD and stress, post-traumatic stress disorder, depression and suicide. This will generate sufficient evidence to design tailored intervention for police officers as an important group within the community in terms of enforcing the laws and as role models for appropriate behavior [19].

A strength in this study is that it is the only study done to assess the magnitude and factors associated with substance use disorders among Police Officers in Tanzania. This study included Police Officers from all levels, departments and special units so as to enhance generalizability of the findings in terms of organizational and operational factors. Besides the strengths of this study, a number of limitations are noted. Limited studies exist on substance use among Police Officers in Tanzania and hence less information and relevant experience to build on. The use of self-administered questionnaires may have led to a significant number of missing data due to incomplete responses which might have affected the quality of the results. However, this data collection method was preferred over interviews so as to allow confidentiality and transparency among participants. Additionally, the approach was based on self-reports and, since alcohol and illicit substance use are against police code of conduct, the likelihood of underreporting and obtaining socially desirable answers was high. Further, the study was done among Police Officers in Urban setting where operational and organizational factors are different from the rural setting making it challenging to generalize the findings for all Police Officers in Tanzania. Generalization of these findings should be done with caution considering the cut off points use to establish SUD and the cross-sectional design of the study which make it challenging to establish causal relationship. The prevalence for SUD is likely to be elevated due to collapsing of high and moderate risk substance use to represent SUD as majority of the study participants had moderate risk for substance use. However, similar categorizations have been used in previous studies and were validated with high sensitivity and specificity [32].

## Conclusion

Substance use and substance use disorder is found to be common among Police Officers in this study. This calls for comprehensive substance control measures to this subpopulation group. Alcohol and tobacco are found to be the most commonly used substances with about 1 in every 10 police officers having potential AUD and 6 out of 100 having potential TUD. Depression was found to be independently associated with both potential AUD and TUD. However, potential AUD was found to be the only independent factor for potential TUD. Further research should be conducted to look with more detail into the predictors of SUDs among Police Officers. There is a pressing need for conducting screening of substance use among police officers frequently and provide early intervention to those in need. This could go along with formulation of workplace wellness policy guidelines, mental health awareness education and establishment of training programs for medical and social worker staff among Police Officers to identify and provide services to officers with risky substance use patterns.

## Data Availability

Data can be made available upon reasonable request from the corresponding author.

## Abbreviations

AUD: Alcohol Use Disorder
PTSD: Post-Traumatic Stress Disorder
PHQ-9: Patient Health Questionnaire
SUDs: Substance Use Disorders
TUD: Tobacco Use Disorder
